# Methods for measuring body composition in Zambian adolescents living with HIV

**DOI:** 10.1371/journal.pgph.0003200

**Published:** 2024-12-19

**Authors:** Suzanne Filteau, Molly Chisenga, Cassandra Namunkonda, Cynthia Mukwasi-Kahari, Lackson Kasonka, Victoria Simms, Celia L. Gregson, Jonathan C. Wells

**Affiliations:** 1 Faculty of Epidemiology and Population Health, London School of Hygiene and Tropical Medicine, London, United Kingdom; 2 University Teaching Hospital, Lusaka, Zambia; 3 The Health Research Unit (THRU-ZIM), Biomedical Research and Training Institute, Harare, Zimbabwe; 4 Musculoskeletal Research Unit, Bristol Medical School, University of Bristol, Bristol, United Kingdom; 5 Institute of Child Health, University College London, London, United Kingdom; Qatar University College of Medicine, QATAR

## Abstract

Assessment of body composition can be useful in managing many clinical or public health conditions, including HIV. Most people living with HIV infection are in Africa where clinics may lack equipment, utilities or staff time for optimal body composition assessment. Thus, it is important to determine whether less expensive and more available and scalable methods can provide adequate information. We estimated body composition of 420 perinatally HIV-infected Zambian adolescents, aged 11–19 years, using dual-energy X-ray absorptiometry (DXA), bioelectrical impedance (BIA) and anthropometry: body mass index (BMI), waist circumference, and subscapular and suprailiac skinfolds. Data were converted to internal population Z-scores in order to compare measures. BIA and DXA were compared for total fat and fat-free mass, BMI was compared to DXA fat mass, and waist circumference and skinfolds were compared to DXA trunk (central) fat. Neither anthropometry nor BIA adequately reflected total fat or trunk fat as measured by DXA. Although mean bias was generally small, especially for females, the limits of agreement were wide for all comparisons. In addition, for central fat in males, the bias was larger at higher mean Z-score values. BMI had similar agreement with DXA fat mass, as did BIA in females, though not males. We conclude that, because of wide limits of agreement and bias in some measures, none of the simpler methods examined are adequate for assessing longitudinal changes in body composition in order to monitor children’s health. However, where BIA is available and DXA is not, BIA may still be able to describe broad trends in body composition of African adolescents living with HIV.

## Introduction

Of the 2 million children living with HIV globally, 90% live in sub-Saharan Africa. Perinatal HIV infection is associated with several chronic complications in children [1], including impaired early linear growth (stunting) [2, 3]. There is evidence both for [4, 5] and against [6, 7] the idea that stunted children are at higher risk of overweight and its associated metabolic changes later in life. The differences likely result, in part, from different underlying medical conditions and environments, while also reflecting uncertainty regarding whether any association between stunting and overweight reflects biological mechanisms or simply confounding by common exposure to drivers of all forms of malnutrition. Among children and adolescents living with HIV, different antiretroviral therapy (ART) regimens have been associated with both different linear growth patterns and body fat content, emphasising the importance of understanding growth and body composition in perinatally HIV-infected children [2].

Both major components of body composition, i.e. fat and fat-free mass, are associated with overall health and it is important for clinicians to measure these in patients with a variety of illnesses in order to provide appropriate interventions [8]. Multiple tools have been used to assess body composition, all having advantages and disadvantages, different assumptions and different methods for converting raw data into fat and fat-free mass [9]. Since the only true direct measurement of body composition, cadaver dissection, is not feasible in clinical or public health contexts, the 4-component model is considered a gold standard; however, it requires expensive equipment and time which are often not available to those caring for people living with HIV in Africa. Other body composition assessment methods, in descending order of cost and feasibility, are dual-energy X-ray absorptiometry (DXA), bioelectrical impedance assessment (BIA), detailed anthropometry including skinfolds, and simple height and weight to calculate body mass index (BMI). DXA provides data for both total and regional fat and fat-free (i.e. lean plus bone) mass; for this study we were interested in trunk fat as an indicator of central adiposity. However, compared to the 4-component model, DXA shows bias to different degrees, some quite high as a percentage of the mean, which varies depending on the measure considered, the value of the measure, and the population studied [10, 11]. BIA equipment is considerably cheaper to buy and maintain than DXA machines and, importantly, does not use radiation and can be operated by nurses or other clinic personnel after only brief training. BIA was considered adequate for assessing body composition of HIV-infected Ethiopian adults [12] and has been used for perinatally HIV-infected Brazilian adolescents [2]. DXA and BIA use different assumptions and, unfortunately, they do not always agree well with each other [13]. Anthropometry is widely available and cheap and hence BMI is often used to assess over- or under-weight, including for HIV-infected Mozambican children and adolescents [3]. However, although very high BMI generally reflects high body fat and very low BMI reflects low amounts of both fat and fat-free mass, at less extreme BMI levels it is hard to determine the amounts of both fat and fat-free mass. Furthermore, BMI does not provide information about fat distribution although other anthropometric measurements such as waist circumference and skinfold thicknesses can provide such information. Although height, weight and waist circumference seem easy to measure, staff training is required to obtain reliable measurements; skinfold thicknesses especially require more experienced anthropometrists. Furthermore, close contact for detailed anthropometry is not always culturally appropriate so only weight and height assessment may be done.

The aim of this analysis was to explore agreement of body composition assessments using lower cost, scalable, methods—BIA and anthropometry—against DXA values in a population of perinatally HIV-infected adolescents with a high prevalence of stunting. This could enable clinicians in facilities lacking expensive equipment to choose a body composition assessment method suitable for their environment in order to intervene, if needed, to mitigate risks of unhealthy body composition and its metabolic consequences in children whose treatment they manage.

## Materials and methods

### Study design and participants

This cross-sectional study used baseline data from the vitamin D3 and calcium carbonate supplementation for adolescents with HIV to reduce musculoskeletal morbidity and immunopathology (VITALITY) trial, a randomised controlled trial conducted in Zambia and Zimbabwe (Pan African Clinical Trials Registry ID: PACTR20200989766029) [1]. The trial primary outcome was total body less head bone mineral density Z-score for which results will be reported separately. For this analysis, only the Zambian data were used since the Zimbabwean site did not collect detailed anthropometry or body composition data using BIA.

Participants were recruited from Lusaka outpatient HIV clinics between March 1, 2021 and November 23, 2021 and referred for enrolment at the Women and New-born Hospital at the University Teaching Hospital, Lusaka. Inclusion criteria were age 11–19 years, perinatally-acquired HIV infection, taking ART for at least 6 months, having a firm home address and intending to remain there for 96 weeks, having a defined caregiver (for those aged <18 years), being aware of their HIV status (for those aged >12 years), willing to give blood samples and rectal swabs, guardian consent and participant assent for those aged <18 years or participant consent for those aged >18 years. Exclusion criteria were any condition likely to prove fatal during the study period, taking tuberculosis treatment, pregnant or breastfeeding, having a condition likely to lead to lack of understanding of or cooperation with study procedures, history of thyrotoxicosis, lymphoma, renal calculi, chronic renal disease, osteomalacia, hypercalcemia or a disorder of phosphate metabolism, physical or radiological signs of rickets or osteomalacia, acutely unwell and requiring immediate medical attention, living in the same household as a trial participant, high likelihood of non-adherence to trial medication. Further details about the VITALITY trial are available in the protocol paper [1]. HIV-specific data, that is ART regimen and plasma HIV viral load, were taken from clinical records. The lower detection limit for viral load in the local services was 60 copies/μl.

### Ethical and governance approvals

Approvals for the VITALITY trial were obtained from the University of Zambia Biomedical Research Ethics Committee, the National Health Research Authority of Zambia, the Medical Research Council of Zimbabwe, the Biomedical Research and Training Institute Internal Review Board (Zimbabwe), and the Ethics Committee of the London School of Hygiene and Tropical Medical. Participants over 18 years provided written informed consent. For those under 18 years, their guardian provided written informed consent and the participant provided assent.

### Anthropometry and body composition assessment

All anthropometric measures–weight, height, waist circumference and triceps, subscapular and suprailiac skinfold thicknesses—were taken in triplicate using standard methods [14] following a period of training. The range of the three measures was checked to look for outliers which were corrected or removed when found. Means of the remaining valid measures were used in analyses. Technical error of measurement calculations [15] were performed periodically and agreement among anthropometrists was within acceptable limits (inter-rater difference <0.5 cm for height or waist circumference, <0.9 mm for skinfolds). BMI was calculated as weight in kg divided by height in m^2^.

Body fat and lean proportions, assessed as fat mass and fat-free mass, respectively, by BIA and DXA, were conducted on the same day as anthropometry. For BIA we used a Tanita BC 418 machine (Tanita, Tokyo, Japan) and followed the manufacturer’s instructions. The machine uses internal formulas to calculate fat and fat-free mass and does not provide raw data in impedance. For DXA we used a Hologic QDR Wi model with Apex software version 4.5 (Hologic Inc., Bedford, MA, USA). Before undergoing the DXA scan, participants were asked to confirm that they were able to lie flat on their back, that they had not had any radiology contrast enhanced examination within the past week and that they had removed all radiopaque objects, any clothing with metal fasteners or any metal jewellery. Within the DXA unit, each participant was asked to change into a loose comfortable fitting gown and to remove their shoes. For females, a urine pregnancy test on the day of the DXA scan was used to confirm they were not pregnant. We calculated total fat-free mass as the sum of total lean and bone mass. Daily calibration of the DXA machine was done with the manufacturer-provided spine phantom. All participants were scanned on the same DXA machine. DXA scans were repeated in sixty participants to assess reproducibility and the percent coefficient of variation (% CV) was <2% for lean mass and <3% for fat mass.

### Data handling and statistical analysis

Data were collected on Samsung tablets using electronic forms designed with OpenDataKit (ODK; https://getodk.org/) software. Further cleaning and checking of distributions as well as analyses were done using Stata 18 (College Station, USA). We calculated internal population Z scores of anthropometry and body composition data for two reasons: to control for age and sex, and so that all variables (BIA and DXA measures of FM and FFM in kg, BMI in kg/m^2^, waist circumference in cm, skinfolds in mm) would be on a common scale for comparisons by Bland-Altman analysis. We used the LMS (lambda, mu, sigma) method [16] which generates three parameters (LMS for power, mean and standard deviation) for each month age band and then calculated individual Z scores using these parameters in combination with individuals’ measurements. In addition, for comparison with an external reference, anthropometric Z scores were calculated for BMI-for-age (BMIZ), height-for-age (HAZ), and waist circumference-for age (WCZ) using the Stata zanthro commands and UK reference values [17]. The UK growth references were used for consistency in the VITALITY study since the references for bone density by DXA, the primary outcome in the trial, are derived from UK populations.

We used scatterplots to illustrate associations between continuous measures and Bland-Altman plots to assess agreement and bias between methods of assessing fat and fat-free mass. We also investigated correlations between the mean of two measures and their bias, to ascertain if agreement between methods varies with the magnitude of the trait (fat mass or fat-free mass). Total body DXA was our main reference for total fat and fat-free mass by BIA. Trunk fat by DXA was the reference for central adiposity which was compared with waist circumference and subscapular and suprailiac skinfolds Z-score; these low-cost methods are associated with central adiposity but do not directly asess it. We explored comparisons of BIA data with total-body-less-head (TBLH) fat and fat-free mass by DXA out of concern that intermittent use of hair pieces by girls may affect DXA body composition assessment, given that hair might have a similar density to fat or fat-free mass. (N.B. DXA software is able to identify a region of interest that represents the head, and subtract values from the total body measurements–this is the standard approach for paediatric DXA [18]). We also investigated the association between BMI and total body fat mass by DXA, since BMI is widely used clinically. Analyses were exploratory and significance testing was not conducted.

## Results

A flow chart showing selection of participants is in S1 Fig. Approximately half of the 420 adolescents recruited were female and the age distribution was approximately equal across age bands (Table 1). Participants had been on ART for on average 6.7 years, mostly Tenofovir Disoproxil Fumarate-based regimens, yet 16% still presented with high HIV viral loads. Fewer than half the participants had both parents still living. Males were fairly evenly distributed across Tanner stages based on testes size, whereas most girls were in higher Tanner stages based on breast size.

**Table 1:** Description of study population, N = 420.

Sex (#, % female)	230 (55%)
Age group, years (#, %)	
11–13	100 (24%)
13–15	110 (26%)
15–17	91 (22%)
17–19	119 (28%)
Tanner stage, males (#, %)	
1	15 (8%)
2	54 (28%)
3	47 (25%)
4	40 (21%)
5	34 (18%)
Tanner stage, breasts, females (#, (%)	
1	16 (7%)
2	18 (8%)
3	43 (19%)
4	62 (27%)
5	91 (40%)
Age started ART,^1^ years (mean, SD)	6.7 (4.3)
ART^1^ regimen (#, %)	
Tenofovir Disproxil Fumarate	358 (85%)
Azidothymidine	50 (12%)
Abacavir	11 (3%)
HIV viral load, copies/μl (#, %)	
<60	354 (84%)
> = 60 & <1000	25 (6%)
> = 1000	41 (10%)
Parental survival (#, %)	
Both known alive	202 (48%)
One parent known to be dead	144 (34%)
Both parents known to be dead	60 (14%)
Missing data	14 (3%)
Mother’s highest education level (#, %)	
None	20 (5%)
Primary	131 (31%)
Secondary	163 (39%)
College/university	13 (3%)
Missing	93 (22%)
Father’s highest education level (#, %)	
None	6 (1%)
Primary	37 (9%)
Secondary	150 (36%)
College/university	53 (13%)
Missing	174 (41%)
Housing (#, %)	
Own home	184 (44%)
Rent main dwelling	39 (9%)
Rent or use part of dwelling	197 (47%)

^1^ ART = antiretroviral therapy; ART regimen groups refer to the main nucleoside reverse transcriptase inhibitor.

Anthropometric summary statistics and Z scores against the UK reference population are shown in Table 2. As indicated by Z scores, males generally had greater anthropometric deficits, compared to the UK reference, than females. Fat mass was lower when assessed by BIA than by DXA but both fat and fat-free mass by the different methods were highly associated (S2 Fig). Excluding the head removed about 3.5 kg of fat-free mass and about 1 kg of fat mass from DXA body composition estimates in both sexes.

**Table 2:** Anthropometry and body composition of study participants ^1^^,^ ^2^^,^ ^3^.

	Males (n = 190)	Females (n = 230)
Weight (kg)	40.5 (9.5)	44.3 (10.0)
Height (cm)	153.1 (12.1)	151.3 (9.1)
HAZ	-1.66 (1.02)	-1.19 (1.04)
BMI (kg/m^2^)	17.0 (1.9)	19.1 (2.9)
BMI Z	-1.35 (0.97)	-0.44 (1.03)
Waist circumference (cm)	63.3 (5.5)	66.7 (6.8)
WCZ	-0.96 (0.87)	0.36 (0.98)
Subscapular skinfold (mm)	6.2 (5.1, 7.3)	9.7 (7.5, 12.6)
Suprailiac skinfold (mm)	4.9 (4.1, 5.7)	7.5 (5.9, 10.0)
BIA body composition		
Fat mass (kg)	5.6 (4.7, 6.8)	10.1 (7.4, 12.4)
Fat-free mass (kg)	34.2 (8.3)	33.5 (6.3)
DXA body composition		
Fat mass (kg)	7.0 (5.8, 8.4)	11.7 (8.9, 14.7)
Fat-free mass, including bone (kg)	34.3 (8.6)	32.9 (6.2)
Bone mineral (kg)	1.3 (0.4)	1.4 (0.3)
Trunk fat mass (kg)	2.5 (2.0, 3.1)	4.3 (3.1, 5.4)
TBLH fat mass (kg)	6.0 (4.9, 7.4)	10.7 (8.1, 13.7)
TBLH fat-free mass (kg)	30.6 (8.2)	29.2 (5.9)
TBLH bone mineral (kg)	1.0 (0.3)	1.1 (0.3)

^1^ BIA, bioelectrical impedance; BMI, body mass index; BMIZ, BMI Z score; DXA, dual X-ray absorptiometry; HAZ, height-for-age Z score; TBLH, total body less head; WCZ, waist circumference Z score

^2^ Values are mean (SD) except for fat masses in kg and skinfolds which were log-normally distributed so medians (25^th^, 75^th^ percentiles) are presented.

^3^ Z scores for height, BMI and waist circumference were calculated using zanthro in Stata and the UK growth reference.

**Fig 1:**
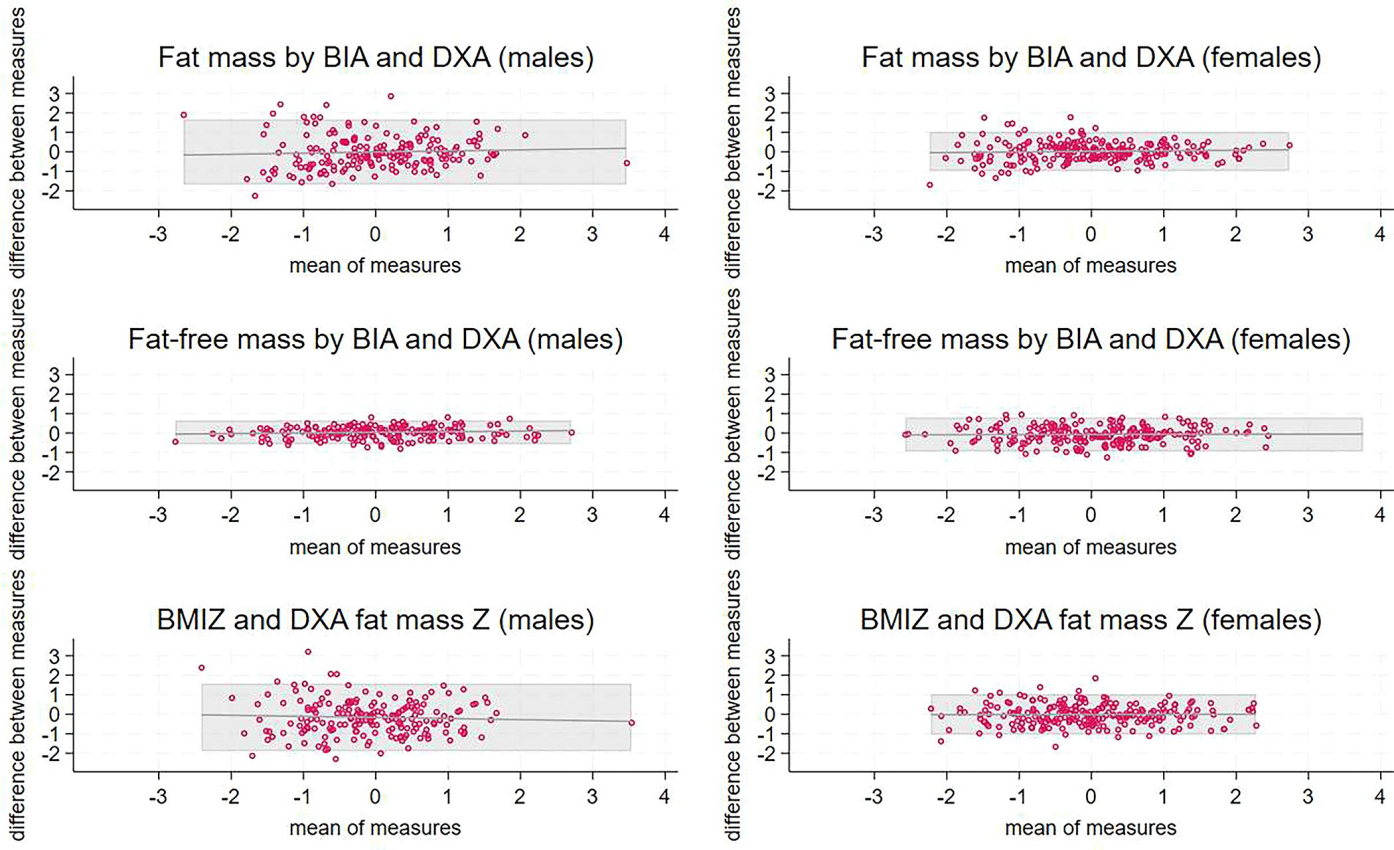
Bland-Altman plots comparing Z scores for total fat and fat-free mass by BIA and DXA. ^1^ X axes are means of the two Z scores and Y axes are the differences between the two. BIA and DXA are measures of fat and fat-free mass whereas BMI is a proxy indicator of fat mass. Shaded areas represent limits of agreement. Lines indicate the trend lines for the comparisons. ^2^ A, B) total fat mass Z by BIA and DXA for males, females; C, D) total fat-free mass Z by BIA and DXA for males, females; E, F) body mass index Z versus fat mass by DXA for males, females. ^3^ BIA, bioelectrical impedance; BMI, body mass index; DXA, dual X-ray absorptiometry.

**Fig 2:**
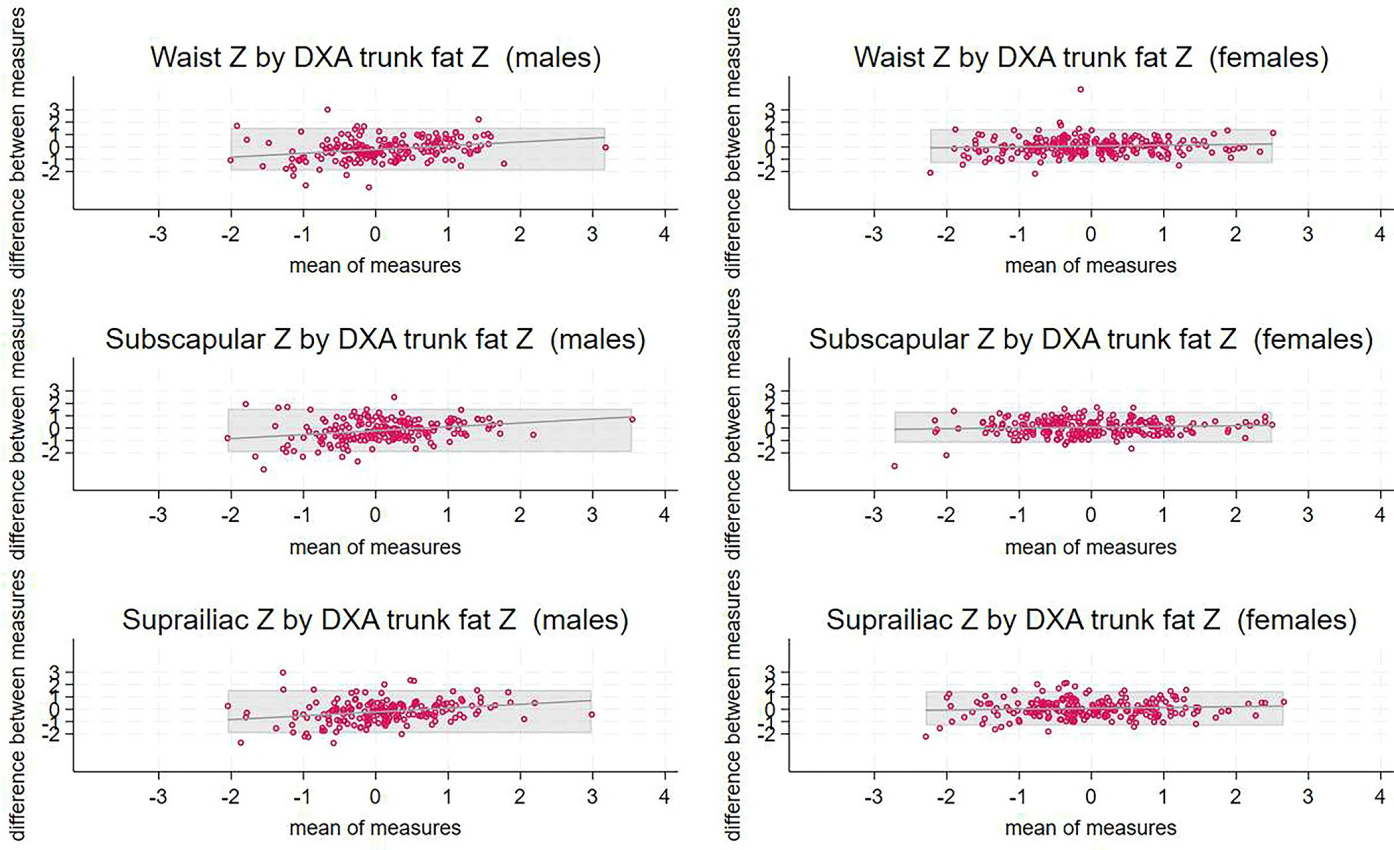
Bland-Altman plots comparing anthropometric Z scores associated with central fat and trunk fat Z score by DXA. ^1^ X axes are means of the two Z scores and Y axes are the differences between the two. DXA provides a measure of trunk fat whereas the anthropometric variables are proxy indicators of fat mass. Shaded areas represent limits of agreement. Lines indicate the trend lines for the comparisons. ^2^ A, B) waist circumference and trunk fat mass by DXA for males, females; C, D) subscapular skinfold and trunk fat mass by DXA for males, females; E, F) suprailiac skinfold and trunk fat mass by DXA for males, females. ^3^ DXA, dual X-ray absorptiometry.

Bland-Altman plots showing comparisons between methods for total body fat or fat-free mass are shown in Fig 1, plots comparing methods for central fat are in Fig 2, and scatterplots showing the associations between pairs of body composition indicators are in S3 Fig. Results for TBLH DXA comparisons with BIA were very similar to the results including the head; plots are shown in S4 Fig. For all comparisons in females, the mean bias was small, although was different from zero for fat-free mass by BIA versus DXA, and the bias was not associated with the mean; however, the limits of agreement were wide (Table 3). For males, methods compared less well: the mean bias was generally large and the bias was greater at higher values for comparisons of central fat, i.e. skinfolds and waist circumference, with trunk fat by DXA.

**Table 3:** Agreement between methods for assessing fat and fat-free mass Z scores^1^^,^^2^.

	Mean difference / bias (95% confidence interval)	Limits of agreement from Bland-Altman analysis	Coefficient from regression equation of bias with mean (95% confidence interval)
Males			
Total fat mass by BIA versus DXA	-0.009 (-0.132, 0.115)	-1.68, 1.67	0.057 (-0.084, 0.197)
Total fat-free mass by BIA versus DXA	0.038 (-0.008, 0.083)	-0.58, 0.66	0.033 (-0.014, 0.079)
BMI versus DXA total fat	-0.162 (-0.289, -0.035)	-1.90, 1.58	-0.56 (-0.209, 0.097)
Waist circumference versus DXA trunk fat	-0.177 (-0.305, -0.050)	-1.92, 1.57	0.307 (0.150, 0.465)
Subscapular skinfold versus DXA trunk fat	-0.181 (-0.310, -0.053)	-1.94, 1.58	0.316 (0.158, 0.474)
Suprailiac skinfold versus DXA trunk fat	-0.186 (-0.314, -0.058)	-1.94, 1.56	0.310 (0.151, 0.468)
Females			
Total fat mass by BIA versus DXA	0.025 (-0.043, 0.093)	-0.98, 1.03	0.031 (-0.041, 0.102)
Total fat-free mass by BIA versus DXA	-0.076 (-0.135, -0.017)	-0.95, 0.80	0.006 (-0.055, 0.067)
BMI versus DXA total fat	-0.012 (-0.081, 0.057)	-1.06, 1.03	0.005 (-0.069, 0.079)
Waist circumference versus DXA trunk fat	0.073 (-0.020, 0.165)	-1.32, 1.46	0.066–0.036, 0.168)
Subscapular skinfold versus DXA trunk fat	0.070 (-0.014, 0.154)	-1.20, 1.34	0.061 (-0.030, 0.153)
Suprailiac skinfold versus DXA trunk fat	0.074 (-0.020, 0.167)	-1.33, 1.48	0.069 (-0.034, 0.172)

^1^ BIA, bioelectrical impedance assessment; BMI, body mass index; DXA, dual X-ray absorptiometry by Hologic

^2^ Data were analysed as internal Z scores, with whole body DXA used as the reference for total fat or fat-free mass and trunk fat DXA as the reference for central fat assessed by skinfolds or waist circumference. Coefficients are from linear regression of bias as a function of the mean. Limits of agreement are from Bland-Altman analyses.

## Discussion

In a population of adolescents with perinatal HIV infection, neither anthropometry nor BIA Z-scores adequately reflected total fat or fat-free mass Z-scores or trunk fat Z-scores as measured by DXA. Although mean bias was generally small, especially for females, the limits of agreement were wide for all comparisons. In addition, for central fat in males, the bias was larger at higher mean Z-score values. BMI had similar agreement with DXA fat mass as did BIA fat mass in females, though not males, possibly because a greater proportion of females’ BMI is fat mass, especially since the females were at higher Tanner stages than the males.

At the level of the individual, much of the wide limits of agreement may have a methodological basis. For example, DXA is especially prone to error in the trunk region [9]. The principle of DXA is that it first assigns all pixels to bone versus non-bone, and then evaluates the ratio of lean soft tissue to fat in non-bone pixels. This method results in lean individuals providing few non-bone pixels for soft tissue assessment, whereas individuals with high adiposity supply a large number of pixels with high fat content. Overall, this leads to the underestimation of trunk fat in lean individuals and its overestimation in those with higher body fat [19]. This issue may have contributed to the poor and biased associations of anthropometry and central fat in these males who, overall, had low central fat, as indicated by the low waist circumference Z-score based on the UK standards.

All methods of assessing body composition have their limitations. We used DXA as a comparison method but it is known to deviate, sometimes widely, from the gold standard 4-component model depending on the population or disease state [11]. Furthermore, DXA machines are expensive, not widely available in clinics treating adolescents with HIV, and require highly trained staff. BIA machines are cheaper, more available and easier to use; however, many commonly used models use internal equations derived from populations which may not reflect the population of interest in terms of limb and trunk proportions [20]. Although anthropometry is relatively cheap and easy, skinfold thicknesses look only at individual fat depots and waist circumference includes organs as well as fat which may partly explain the poor associations seen, especially in males. Furthermore, the fat content of the trunk relates to both tissue depth and trunk height; DXA assesses both components, whereas waist girth and skinfold thicknesses are agnostic to trunk height in young people, although this may change in elderly people with vertebral fractures. Nevertheless, in many situations waist circumference can be considered a useful proxy for abdominal fat [9].

Although BIA may be acceptable for assessing body composition of adolescents living with HIV in some clinical situations or cross-sectional studies, it may not be good enough to monitor changes in individual risks of having excess or insufficient total fat. This is especially the case for males since the bias was associated with the mean of the measures. Many basic BIA machines available in Africa do not provide information on trunk versus peripheral fat and fat-free mass. Thus, in many places it will be difficult to obtain, using BIA, important information regarding central fat which carries the largest risk of chronic diseases or of lower body fat which may help protect against chronic diseases [21].

A strength of the study is its detailed assessment, conducted by an experienced research team, of anthropometry and body composition using several methods in a fairly large number of perinatally HIV-infected African adolescents, a group which is understudied. The study also has several limitations. We did not have the full 4-component model but used DXA as a comparison for other methods. Secondly, we did not have access to BIA equipment which could provide either raw impedance data or segmental results.

In conclusion, since body composition assessment can help with clinical management of children living with HIV, it should be done with the best available method, e.g. DXA. Though not agreeing well with DXA, basic BIA equipment can still provide information about broad trends in body composition of African adolescents who are living with HIV or who are stunted. However, where even BIA is unavailable, measurement of BMI would still provide some information about adolescent body composition, growth and health and may help with clinical management. The anthropometric measurements used as proxies for central adiposity did not agree well with the reference method and we do not recommend them for clinical management in a population such as we studied.

## Supporting information

S1 Checklist Inclusivity in global research. (DOCX)

S1 Fig Flow chart of participant recruitment to Zambian arm of VITALITY trial. (DOCX)

S2 Fig Comparison between DXA and bioelectrical impedance of fat mass and fat-free mass in kg. (DOCX)

S3 Fig Scatterplots of Z scores for fat mass, fat-free mass, and anthropometry compared to DXA. (DOCX)

S4 Fig Bland-Altman plots comparing Z scores for total fat and fat-free mass by BIA and with the total body less head DXA measures. (DOCX)
