# Supplementary material for: Methods for measuring body composition in Zambian adolescents living with HIV
Source: PLOS Glob Public Health. 2024 Dec 19;4(12):e0003200. doi: 10.1371/journal.pgph.0003200 (PMC11658486; doi:10.1371/journal.pgph.0003200)
Supplement: S1 Fig — (DOCX) [file pgph.0003200.s002.docx]

**S Fig. Flow chart of participant recruitment to Zambian arm of VITALITY trial**

Screened

(age 11-19)

765

Eligible

420

Enrolled

420

Refused 0

Not eligible

No defined guardian 59

On ART < 6 months 78

No fixed address 62

HIV diagnosis not disclosed to child 13

Not perinatal HIV 15

Sibling enrolled 37

Current TB or TB treatment 57

Pregnant/breastfeeding 22

On supplement 1

Allergic 1
