# Supplementary material for: Methods for measuring body composition in Zambian adolescents living with HIV
Source: PLOS Glob Public Health. 2024 Dec 19;4(12):e0003200. doi: 10.1371/journal.pgph.0003200 (PMC11658486; doi:10.1371/journal.pgph.0003200)
Supplement: S2 Fig — (DOCX) [file pgph.0003200.s003.docx]

**S2 Fig. Comparison between DXA and bioelectrical impedance of fat mass and fat-free mass in kg**

FM_BIA=0.83*FM_DXA + 0.10

FFM_BIA=0.96*FFM_DXA + 1.65

Text boxes show regression equations. FM_BIA and FM_DXA are fat mass by BIA and DXA; FFM_BIA and FFM_DXA are fat-free mass by BIA and DXA; BIA, bioelectrical impedance; DXA, dual X-ray absorptiometry
