# Supplementary material for: Methods for measuring body composition in Zambian adolescents living with HIV
Source: PLOS Glob Public Health. 2024 Dec 19;4(12):e0003200. doi: 10.1371/journal.pgph.0003200 (PMC11658486; doi:10.1371/journal.pgph.0003200)
Supplement: S3 Fig — (DOCX) [file pgph.0003200.s004.docx]

**S3 Fig. Scatterplots of Z scores for fat mass, fat-free mass, and anthropometry**

1. **B.**

β=0.89 (0.82. 0.96)

β=0.65 (0.53, 0.77)

1. **D.**

β=0.91 (0.85, 0.96)

β=0.98 (0.93, 1.03)

**E F**

β=0.88 (0.82, 0.95)

β=0.64 (0.53, 0.76)

**G H**

β=0.89 (0.84, 0.95)

β=0.99 (0.94, 1.03)

1. **J.**

β=0.86 (0.79, 0.93)

β=0.53 (0.42, 0.65)

******K. L.**

β=0.78 (0.69, 0.87)

β=0.67 (0.51, 0.82)

**M. N.**

β=0.82 (0.74, 0.91)

β=0.66 (0.51, 0.82)

**O. P.**

β=0.78 (0.68, 0.87)

β=0.66 (0.51, 0.82)

^1^ Numbers within graphs are regression coefficients and 95% confidence intervals for the comparisons

2 A, B) fat mass Z by BIA and DXA for males and females; C, D) fat-free mass Z by BIA and DXA for males and females; E, F) fat mass Z by BIA and TBLH DXA for males and females; G. H) fat-free mass Z by BIA and TBLH DXA for males and females; I, J) body mass index Z and DXA fat mass Z for males and females; K, L) waist circumference Z and DXA trunk fat Z for males and females; M, N) subscapular skinfold Z and DXA trunk fat Z for males and females; O, P) suprailiac skinfold Z and DXA trunk fat Z for males and females.

^3^ BIA, bioelectrical impedance; BMI, body mass index; DXA, dual X-ray absorptiometry; TBLH, total body less head
