# Supplementary material for: Methods for measuring body composition in Zambian adolescents living with HIV
Source: PLOS Glob Public Health. 2024 Dec 19;4(12):e0003200. doi: 10.1371/journal.pgph.0003200 (PMC11658486; doi:10.1371/journal.pgph.0003200)
Supplement: S4 Fig — (DOCX) [file pgph.0003200.s005.docx]

**S4 Fig. Bland-Altman plots comparing Z scores for total fat and fat-free mass by BIA and with the total body less head DXA measures**

**A B**

**C D**

^1^ X axes are means of the two Z scores and Y axes are the differences between the two. Shaded areas represent limits of agreement.

^2^ A, B) total fat mass Z for males, females; C, D) total fat-free mass Z for males, females

^3^ BIA, bioelectrical impedance; DXA, dual X-ray absorptiometry; TBLH, total body less head
